# Mesenchymal-to-epithelial transition of intercalating cells in *Drosophila* renal tubules depends on polarity cues from epithelial neighbours

**DOI:** 10.1016/j.mod.2010.04.002

**Published:** 2010-07

**Authors:** Kyra Campbell, Jordi Casanova, Helen Skaer

**Affiliations:** a Institut de Biologia Molecular de Barcelona-CSIC, Parc Cientific de Barcelona, 08028 Barcelona, Spain; b Institut de Recerca Biomèdica de Barcelona, Parc Cientific de Barcelona, 08028 Barcelona, Spain; c Department of Zoology, University of Cambridge, Downing Street, Cambridge CB2 3EJ, UK

**Keywords:** Mesenchymal-to-epithelial transition, *Drosophila*, Cell polarity, Cell interaction, Renal tubules

## Abstract

The intercalation of mesenchymal cells into epithelia, through mesenchymal-to-epithelial transition (MET), underlies organogenesis, for example, in nephrogenesis, and tissue regeneration, during cell renewal and wound repair. Despite its importance, surprisingly little is known about the mechanisms that bring about MET in comparison with the related and much-studied, reverse process, epithelial-to-mesenchymal transition (EMT). We analyse the molecular events that regulate MET as stellate cells integrate into the established epithelium of the developing renal tubules in *Drosophila*. We show that stellate cells polarise as they integrate between epithelial principal cells and that the normal, localised expression of cell polarity proteins in principal cells is required for stellate cells to become epithelial. While the basolateral and apical membranes act as cues for stellate cell polarity, adherens junction integrity is required to regulate their movement through the epithelium; in the absence of these junctions stellate cells continue migrating into the tubule lumen. We also show that expression of basolateral proteins in stellate cells is a prerequisite for their ingression between principal cells. We present a model in which the contacts with successive principal cell membrane domains made by stellate cells as they integrate between them act as a cue for the elaboration of stellate cell polarity. We suggest that the formation of zonula adherens junctions between new cell neighbours establishes their apico-basal positions and stabilises them in the epithelium.

## 1 Introduction

During embryonic development diverse cell types migrate over large distances and merge with other cell populations to form complex organs. A fundamental requirement that underpins this process is the capacity of a cell to switch between mesenchymal and epithelial states. When mesenchymal, cells are not stably polarised but exhibit motile and adhesive properties that facilitate migration. However, to contribute to epithelial structures, cells must convert to an epithelial state, which involves making adhesive intercellular contacts with neighbouring cells, and establishing apico-basal polarity with respect to their new position in the developing organ.

The transition of cells from mesenchymal-to-epithelial, and their integration into epithelial tissues, must be tightly controlled and precisely coordinated with the behaviour of surrounding cells and tissues. For example, nephrons of the kidney develop via the recruitment of mesenchymal cells to the ureteric buds. These cells undergo MET to form the renal tubules (Saxon, 1987). Failure of cells to undergo MET correctly can lead to development of the pediatric kidney malignancy, Wilms’ tumor (Li et al., 2002). Currently, the mechanisms that normally act to ensure that the integration and MET of mesenchymal cells occurs correctly, are poorly understood. Studies of cells undergoing the reverse process, EMT, have revealed a tight correlation between a decrease in the levels of E-Cadherin (E-Cad) and adhesive junctions, and loss of apico-basal polarity, with a gain in cell motility (reviewed in Moreno-Bueno et al. (2008). This suggests that polarity and adhesion may act to restrict cell motility and retain cells within an epithelium.

We investigated this question in the renal tubules of *Drosophila*, where the ability of cells to undergo MET is central to the development of this tissue. The tubules originate as four epithelial buds that evert from the hindgut, at its junction with the midgut, and increase in size first by cell division and later by cell rearrangement and cell growth. These epithelial cells make up the main cell type in the renal tubules, the principal cells, which are polarised and linked by adherens junctions throughout renal morphogenesis (Campbell et al., 2009). As the tubules develop, a second mesenchymal population of cells is recruited to the tubules from the caudal visceral mesoderm (Denholm et al., 2003). These cells integrate into the epithelial tubules, undergo MET and differentiate into a physiologically distinct subset of tubule cells, the stellate cells. The stellate cells occupy the more distal secretory region of the tubules, mediating chloride and the majority of water fluxes in the formation of primary urine (O’Donnell et al., 1996). Here, we describe how the integration and patterning of stellate cells, in terms of their acquisition of apico-basal polarity and development of intercellular junctions, is coordinated with morphogenesis of the renal tubules. We show that adherens junction formation and the expression of apico-basal polarity cues in both cell types are critical for the correct integration and MET of stellate cells. In an interesting comparison with the process of EMT, polarity and adhesion are shown to be key regulators of MET.

## 2 Results and discussion

### 2.1 Stellate cells integrate into the renal tubules during stage 13 and are separated from each other by convergent-extension movements

**Fig. 1:**
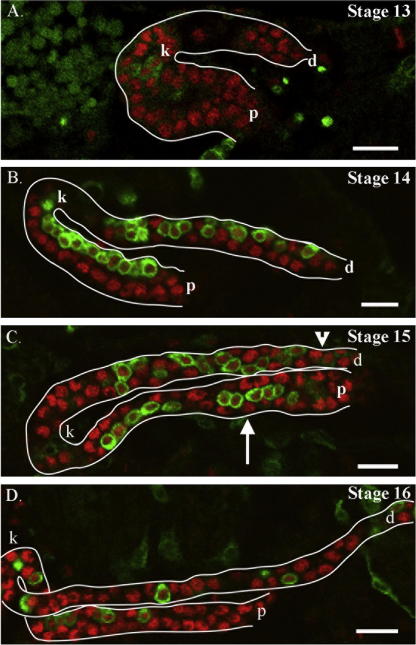
Stellate cells integrate into the renal tubules during stage 13–14 and become spaced out as the tubules elongate. Embryos in which a membrane-bound GFP is driven by G447.2 to visualise the stellate cells, stained for GFP (green) and Ct (red). (A) In early stage 13 embryos only a few stellate cells have integrated into the tubules, and these are clustered on the inner face of the tubule kink. (B) By stage 14 the majority of stellate cells have integrated into the tubules but are not evenly distributed along the tubules, being more spaced out at the distal tip and remaining clustered in the kink and proximal region. (C) In stage 15 the stellate cells are spaced out distally, where the tubule has narrowed (arrowhead), but are still clustered more proximally, where the tubule is thicker in circumference (arrow). (D) By stage 16 the stellate cells are spaced out along the tubules. (k) tubule kink, (p) proximal region, (d) distal region, white lines outline the tubules. Scale bars are 10 μm.

Previous work has shown that the stellate cells integrate into the tubules during stages 13–15 of embryogenesis (Denholm et al., 2003). In order to assess precisely when the integration of the stellate cells takes place, and to understand the behaviour of stellate cells during renal tubulogenesis, we labelled stellate cells with a membrane-bound GFP using G447.2, a Gal4 line that positively marks stellate cells (Denholm et al., 2003). Stellate cells were first detected in the tubules during stage 13, when the renal tubules are curved structures, with the anterior tubules folding back on themselves roughly 2/3 along their length, forming a ‘kink’. It is on the inner face of the kink region that stellate cells are first found during early stage 13 (Fig. 1A). By mid-late stage 14 the process of stellate cell intercalation into the tubules is complete. While those found in the kink region, and just proximal to the kink remain clustered together, stellate cells found more distally in the tubules tend to be separated by principal cells (Fig. 1B). During stages 15 and 16 the proportion of stellate cells separated by principal cells increases, so that they are found increasingly spaced out along the future secretory domain of the tubules (Fig. 1C and D).

**Fig. 2:**
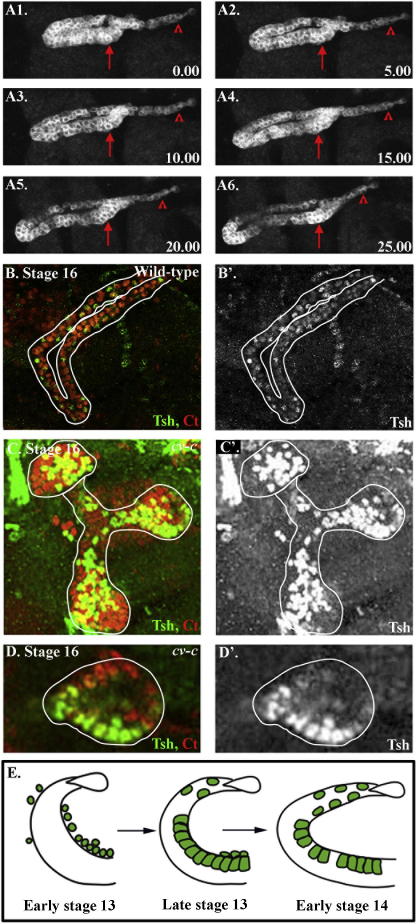
Convergent-extension movements are linked to the mixing of stellate and principal cells. (A) Convergent-extension movements occur first in the distal region of the renal tubules, and move more proximally as the tubules elongate. Stills taken every 5.00 min from a time-lapse movie of elongating tubules in embryos expressing UAS-Src-GFP under CtB-GAL4 control. (A1–A6; Supplementary movie S1). Arrows point to the proximal region and arrowheads to the distal tip. (B–D) Stage 16 wild-type (B) and *cv-c* mutant embryos (C and D) stained for Tsh (green) to visualise the stellate cells and Ct (red). (B) In wild-type stage 16 embryos the tubules are long, thin tubes with stellate cells spaced out along their length. (C) In stage 16 *cv-c* mutants, the tubules appear as short, thick structures, due to the failure of convergent-extension movements. A 25 μm z-stack through a *cv-c* mutant embryo, shows that stellate cells are clustered together in the tubule epithelium. (D) A single confocal z-section of a *cv-c* mutant tubule confirms that stellate cells remain clustered together and do not mix with principal cells. (E) Model illustrating the behaviour of stellate cells during stages 13 and 14. They integrate into the tubules during stage 13 and are spaced out as the tubule elongates. White lines outline the tubules.

The progressive separation of stellate cells occurs during stages 13–16, which is also when the tubules undergo elongation by convergent-extension movements, suggesting that there may be a correlation between these two events. During convergent-extension movements, the tubules decrease in circumference from an 8 to 12 cell diameter to a 2 cell diameter, and extend by four times in length (B. Denholm, personal communication). Interestingly, these movements do not take place uniformly along the length of the tubules, occurring first in the distal region and moving more proximally as the tubule extends (Fig. 2A, Supplementary movie S1). Strikingly, the degree to which the stellate cells have separated during stages 14 and 15 correlates with the reduction in tubule thickness (Fig. 1C and D), which also strongly suggests a link between convergent-extension movements and the spacing-out of stellate cells.

To investigate a possible role for convergent-extension movements in the separation of stellate cells, we assessed the distribution of stellate cells in the renal tubules in mutants in which these movements do not take place. *crossveinless-c* (*cv-c*) is expressed in tubule cells and is required for the cell rearrangements that underpin convergent-extension movements (Denholm et al., 2005). In embryos carrying mutations in *cv-c*, the renal tubules do not elongate, but remain short and thick (Fig. 2C (Denholm et al., 2005). In stage 16 *cv-c* embryos stellate cells do not mix with principal cells but instead remain clustered together (cf. Fig. 2B with C and D)*.* Although we cannot rule out a requirement for *cv-c* in stellate cells for normal integration, these data further support the hypothesis that convergent-extension movements drive the separation of stellate cells, and are required for the correct positioning of the cells along the length of the tubules (Fig. 2E).

### 2.2 After integrating into the renal epithelium stellate cells rapidly establish apico-basal polarity and adherens junctions

During MET a mesenchymal cell establishes apico-basal polarity and develops adherens junctions, switching to an epithelial state. During renal tubule development stellate cells transform from mesenchymal-to-epithelial, so that in the mature tissue stellate cells are fully polarised epithelial cells, expressing localised markers of apico-basal polarity and developing a distinct actin brush border (Denholm et al., 2003). To establish when stellate cell MET occurs, we investigated their polarity during tubulogenesis by labelling them with a membrane-bound GFP using G447.2 Gal4 and analysing the expression of cell polarity genes in labelled cells throughout embryogenesis.

**Fig. 3:**
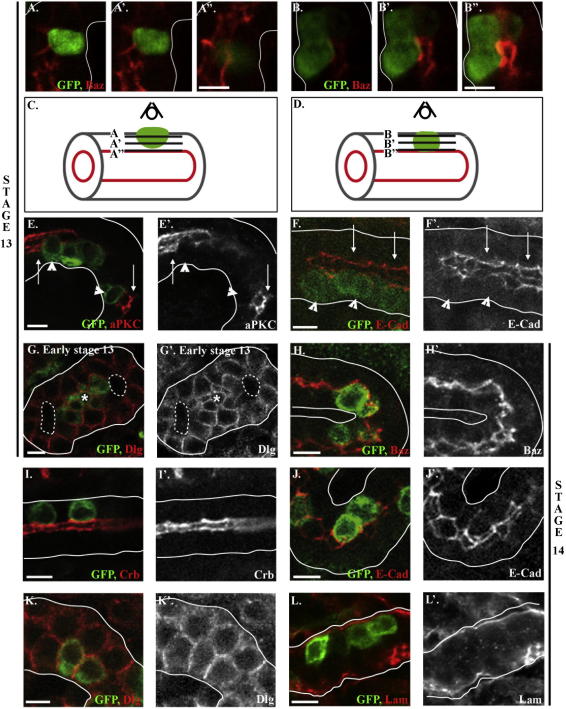
Stellate cells become fully polarised during stages 13 and 14. Embryos in which a membrane-bound GFP is driven by G447.2 to visualise the stellate cells, stained for GFP (green) and polarity proteins (red). (A–D) Sequential confocal z-sections, 1.5 μm thick, moving from the outside to the lumen of the tubule, were used to analyse the expression of Baz in stellate cells of stage 13 embryos. Stellate cells that do not touch the tubule lumen, do not express Baz (A and C), whereas stellate cells that contact the apical surface of their neighbours express and localise Baz (B and D). (E and F) aPKC (E) and E-Cad (F) are also expressed in stellate cells that have just contacted the tubule lumen (arrowheads), though the level of expression of these proteins is lower in stellate cells than in neighbouring principal cells (arrows). (G) Dlg is expressed in stellate cells in early stage 13 embryos, just after they have pushed into the renal epithelium (asterisk labels a stellate cell expressing Dlg). (H–L) In mid-stage 14 embryos, all stellate cells contact the luminal surface of the tubules and express high levels of localised Baz (H), Crb (I), E-Cad (J), Dlg (K). Lam is found on the basal surface of both stellate and principal cells (L). All confocal images are a single z-section through a renal tubule, white lines outline the tubules and dotted lines the lumen. Scale bars are 5 μm.

The process whereby stellate cells integrate into the renal tubules during stage 13 and early stage 14 involves the cells adhering to the outside of the tubules and pushing into the renal epithelia. They move between neighbouring cells until the leading edge of the cell touches the apical/luminal surfaces of their neighbours, at which point they stop moving and become part of the developing tubule. In stage 13 embryos, when stellate cells are first found in the renal tubules, apical and junctional proteins are not detectable in stellate cells whose apical tips have not yet contacted the luminal surface (Fig. 3A and C). However, examination of polarity genes in stellate cells that have reached the lumen revealed that the majority of these cells show localised expression of apical and junctional proteins (Fig. 3B–F), though the levels of these proteins are lower than in the neighbouring principal cells (Fig. 3E and F). We next examined the expression of basolateral proteins and found that the expression of Discs large (Dlg) in stellate cells is initiated earlier than apical and junctional proteins. Stellate cells express Dlg weakly as they establish contact with the tubules, and there is clear expression as they integrate into the renal epithelium, regardless of whether they have contacted the apical surface or not (Fig. 3G).

By mid-stage 14 all stellate cells contact the luminal surface of the tubules and have developed polarity; they express high levels of apical, basolateral and junctional proteins, similar to the principal cells, and are covered by the basement membrane component, laminin, on their basal surface (Fig. 3H–L). These data indicate that as stellate cells push into the renal tubules, they express and localise basolateral proteins. Subsequently, as the stellate cells touch the lumen of the renal tubules, they rapidly establish apico-basal polarity and develop adherens junctions.

### 2.3 Apico-basal polarity and adherens junctions are required in principal cells for the normal integration and polarisation of stellate cells

When the stellate cells reach the apical surface of the renal tubules they stop moving through the tube and establish apico-basal polarity and adherens junctions with respect to their position within the epithelium. This suggests that the membrane polarity of the principal cells, in particular their apical and junctional domains, may act as cues that instruct the stellate cells to stop moving and establish polarity. Furthermore, they may also be important for directing the orientation of stellate cell polarity, and the positioning of adherens junctions.

Therefore, we decided to investigate whether polarity and adherens junctions are required in the principal cells for the normal positioning and polarity of stellate cells. We selectively perturbed polarity and adherens junctions in principal cells by overexpressing full-length Crumbs (Crb) using a tubule specific driver; this has previously been shown to cause a delocalisation of polarity proteins and fragmentation of adherens junctions in the renal tubules from stage 13 (Campbell et al., 2009). It should be noted that this driver expresses in both principal and stellate cells; we do not have a principal cell specific driver. However, by means of a driver that is specific to stellate cells we found that the overexpression of Crb does not affect their integration or polarity (data not shown). Thus, the phenotypes elicited by Crb overexpression in the tubules reflects mostly, if not solely, effects in principal cells.

**Fig. 4:**
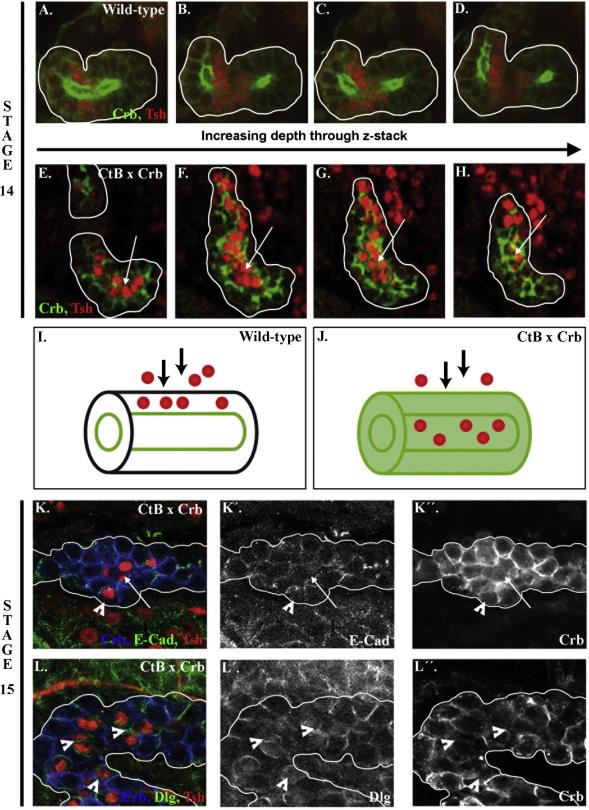
Overexpression of Crb in the renal epithelium disrupts the integration and polarisation of stellate cells. (A and H) Sequential 1.5 μm thick confocal z-sections of renal tubules were used to analyse the positioning of stellate cells in stage 14 wild-type (A–D) and Crb-overexpressing (E–H) tubules stained for Tsh (red) and Crb (green). (A–D and I) In wild-type embryos stellate cells are found around the tubule lumen. (E–H and J) In Crb-overexpressing tubules stellate cells are located in the middle of the tubules in the position of the lumen, arrows in (E–H) point to the lumen. (K–L) Stage 15 Crb-overexpressing tubules stained for Crb (blue), Tsh (red) and E-Cad (K, green) or Dlg (L, green). Stellate cells exhibit varying degrees of polarity, ranging from a complete lack of expression of Crb and E-Cad (K, arrow) to low levels of Crb, E-Cad and Dlg, which are not correctly orientated with respect to the apico-basal axis of the tissue (K, L, arrowheads). White lines outline the tubules.

In wild-type tubules of early stage 14 embryos, stellate cells are located around the tubule lumen, and their apical domains and adherens junctions are in register with neighbouring principal cells (Figs. 3H–J and 4A–D). Remarkably, in embryos in which polarity and adherens junctions have been disrupted in the renal tubules, the stellate cells are located in the middle of the tubules, in the position of the lumen (Fig. 4E–H, arrows). Thus apico-basal polarity and adherens junctions in principal cells are required in the tubule epithelium for the stellate cells to stop moving through the tubule when they reach the apical surface, and to keep contact at their basal side with the outside of the tubule.

We followed the behaviour of the stellate cells in Crb-overexpressing tubules during later stages of development to see if they are able to undergo MET and establish apico-basal polarity according to their position within the renal epithelium. We found that during stages 14 and 15 Crb-overexpressing tubules elongate, changing from a rounded shape to long thick cords of cells. Stellate cells in these tubules exhibit varying degrees of apico-basal polarity. This varies from a complete lack of apical and junctional markers, most apparent when they are clustered together in the middle of the cords of cells (Fig. 4K, arrow), to a low level of expression of apical-basal and junctional proteins (Fig. 4K and L, arrowheads). However, in stellate cells that exhibit some degree of polarisation, the polarity proteins are not orientated with respect to the cell’s position within the tissue, indicating that, while the cells initiate the expression of polarity proteins, they do not localise them correctly.

These data show that apico-basal polarity and the normal development of adherens junctions between epithelial principal cells are required for two features of stellate cell integration and MET. First, contact with the adherens junctions and/or apical domains of polarised principal cells stops the stellate cells moving through the epithelium beyond the correct position. In their absence stellate cells move into the lumen and lose contact with the outside of the tubule. This could be due to the polarised membrane domains on principal cells acting as instructive cues to stop stellate cells moving and to initiate polarity. Alternatively, cell–cell adhesion mediated by the adherens junctions could be acting as a physical restriction to halt migration of the stellate cells.

Second, our results show that polarity and adherens junctions are required in the principal cells for stellate cells to correctly localise polarity and junctional proteins with respect to their position within the tubules. Interestingly, even when stellate cells are mispositioned, they still initiate the expression of polarity proteins. This indicates that stellate cells do not have to reach the correct position within the renal epithelium in order to start the genetic programme that leads to MET, and raises the possibility that contact between stellate cells and principal cells at the tubule surface is sufficient to cause the stellate cells to initiate the switch from mesenchymal-to-epithelial states. However, the correct positioning of stellate cells is clearly required for the full transition to an epithelial phenotype.

### 2.4 Adherens junctions are required for the normal integration of stellate cells, but not for orientating the localisation of polarity proteins

The overexpression of Crb leads to a fragmentation of E-Cad in principal cells, suggesting that the defects we observed in stellate cell integration and polarity could be due to loss of adherens junctions in the principal cells. To investigate further the role of adherens junctions in cell integration and MET, we examined the behaviour of stellate cells in embryos with low levels of E-Cad. We carried out this analysis in strong zygotic mutants for E-Cad, using the allele *shg^G317^*, as embryos completely lacking E-Cad disintegrate early in development, so that it is not possible to analyse the behaviour of cells during mid-embryogenesis (Tepass et al., 1996). Oda and colleagues have shown that E-Cad mRNA and protein are downregulated in mesodermal cells as they undergo EMT during stages 7 and 8, so that after this they lack detectable levels (Oda et al., 1998). Therefore, while in *shg* mutants the principal cells retain some E-Cad of maternal origin, the mesodermal stellate cells completely lack E-Cad as they integrate into the tubules, and thus are not be able to form adherens junctions. Previous work has shown that adherens junctions become disrupted in the principal cells of *shg* mutant embryos at the beginning of stage 13, at the onset of convergent-extension movements, while apico-basal polarity remains relatively unaffected (Tepass et al., 1996; Uemura et al., 1996). Thus, at the time of stellate cell integration, principal cells in *shg* mutants have fragmented adherens junctions but normal apico-basal polarity.

**Fig. 5:**
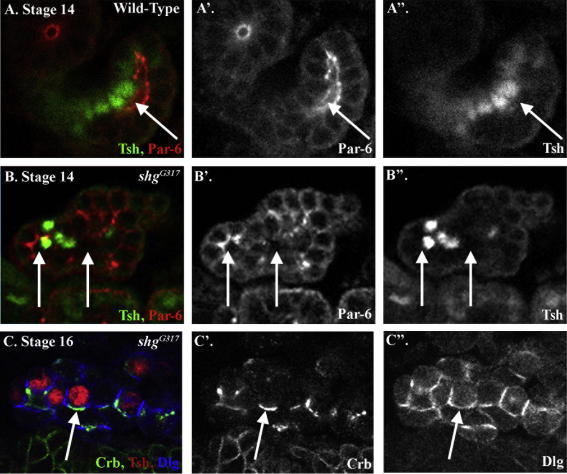
Adherens junctions are required for the correct integration of stellate cells, but not for orienting the axis of polarity. (A and B) Stage 14 embryos stained for Par-6 (red) to visualise the apical surface of tubule cells, and for Tsh (green) to reveal stellate cells, arrows point to the lumen. (A) In wild-type embryos stellate cells are found surrounding the tubule lumen. (B) In *shg* mutant embryos, stellate cells move through the tubules, into the lumen. Arrows point to the lumen. (C) In stage 16 *shg* mutant embryos stellate cells (Tsh, red) localise Crb (green) to their apical surface (arrow) and Dlg (blue) laterally, although it partially overlaps with the Crb expressing domain.

In the renal tubules of stage 14 *shg* mutant embryos, the majority of stellate cells are found in the middle of the tubules, in the position of the lumen (Fig. 5B, compare with wild type, A, arrows point to the position of the lumen). This phenotype is remarkably similar to stage 14 Crb-overexpressing tubules (compare Figs. 5B and 4G). Taken together, these results suggest that it is the adherens junctions, rather than polarised membrane domains, that are required in the principal cells to arrest stellate cell migration and to prevent them continuing into the lumen when they reach the apical surface of the tubule.

To investigate whether adherens junctions are also required for the establishment of polarity in stellate cells, we examined the expression of polarity proteins during later stages of development. During stages 13–16 the renal tubules in *shg* mutants fall apart into small cysts and clumps of tubule cells (Uemura et al., 1996). Examination of remnants of the renal tubules in stage 16 *shg* embryos revealed stellate cells, expressing polarity proteins, lying adjacent to principal cells. Furthermore, apical proteins are localised at the interior face of cells and basolateral proteins, such as Dlg, are largely restricted to their lateral surfaces, although there is some overlap between apical and basolateral proteins that is not seen in wild-type (Fig. 5C). Thus, the localisation of polarity proteins is orientated with respect to the position of the cell in the renal tubule. These data suggest that even in the absence of zygotic E-Cad, where the principal cells of the tubule remnants retain some apico-basal polarity, stellate cells seem able to intercalate and segregate cell polarity proteins to separate membrane domains. However, adherens junctions may be required to reinforce strict separation of apical and basolateral domains.

Our results suggest that as stellate cells integrate into the renal tubules they receive cues from neighbouring epithelial cells, which arrest their movement at a specific apico-basal position, and direct the localisation of cell polarity and junctional proteins, so that they are correctly orientated with respect to the axes already established within the tissue. The polarised membrane domains and adherens junctions of the principal cells appear to play crucial, yet distinct roles during these processes. Adherens junctions are required for stellate cells to stop migrating when the leading edge contacts the lumen of the tubule, whereas polarised principal cell membrane domains are required to orientate the localisation of stellate cell polarity proteins.

It is unclear whether the requirement for adherens junctions is just between principal cells, or if stellate cells need to be able to form junctions in order to stop moving through the tubule. We have attempted to address this question using the tools currently available, such as UAS-E-Cad RNAi lines (see Section 3) and dominant negative E-Cad constructs (Pacquelet and Rorth, 2005) but, as we were unable to achieve significant E-Cad knock-down by stage 13, we have not been able to distinguish between these possibilities (data not shown). Further work will be required to answer these questions, and to determine the mechanisms by which E-Cad and polarised membrane domains act to regulate stellate cell integration and polarity.

### 2.5 Perturbing the establishment of polarity in stellate cells affects their ability to move into the renal epithelium

Stellate cells express and localise polarity proteins as they push into the renal epithelium and move to contact the lumen of the tubule. We reasoned that a feedback loop could be set up as stellate cells begin to express cell polarity genes, whereby cues provided by the polarised domains of principal cells act to reinforce emerging polarity in stellate cells. Thus, early stellate cell polarity would be required for the normal integration of the stellate cells.

To test this, we perturbed polarity in the stellate cells by overexpressing a dominantly active, N-terminally truncated form of aPKC (aPKCΔN, (Betschinger et al., 2003; Drier et al., 2002), using the stellate cell driver, G447.2. Truncated aPKC is no longer restricted to the apical cortex, but becomes active throughout the cell, behaving as a dominantly active form. Expression of this construct should effectively ‘apicalise’ the stellate cells and prevent them from establishing basolateral domains and adherens junctions.

**Fig. 6:**
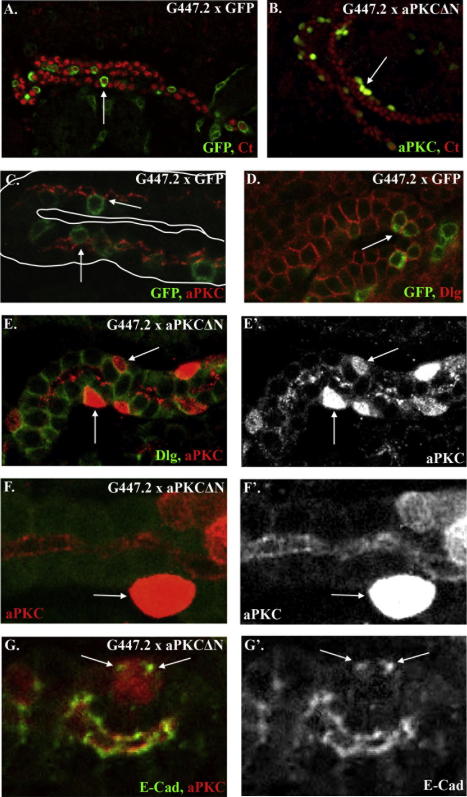
Perturbing the establishment of polarity in stellate cells disrupts their integration. (A, C and D) Embryos in which a membrane-bound GFP is driven by G447.2 to visualise the stellate cells, stained for GFP (green) and Ct (red, A), aPKC (red, C) or Dlg (red, D). (B and E–G) dominant active aPKC-expressing stellate cells stained for (B) aPKC (green) and Ct (red); (E) Dlg (green) and aPKC (red); (F) aPKC (red); (G) E-Cad (green) and aPKC (red). When aPKCN is expressed in stellate cells they do not move into the tubules as in wild-type (compare A with B, arrows). (C and D) In wild-type tubules the stellate cells move into, and touch the tubule lumen (C, arrows) and adopt a columnar morphology (D, arrow). (E and F) When aPKC is dominantly activated in the stellate cells, they do not integrate into the tubules and are abnormal in shape (E and F arrows). (G) Stellate cells that fail to integrate form basal accumulations of E-Cad (G, arrows). All images are of stage 15 embryos. Arrows point to stellate cells.

In wild-type stage 16 tubules, the stellate cells stretch from the basal surface of the tubules to the lumen (Fig. 6A and C). When aPKCΔN is expressed in the stellate cells, they do not move into the renal epithelium but remain on the outside of the tubules (Fig. 6B and E–G). We investigated whether the overexpression of aPKCΔN affects the ability of stellate cells to form adherens junctions with neighbouring cells by staining for E-Cad. Stellate cells in which aPKCΔN has been expressed consistently fail to develop E-Cad junctions with their neighbours but instead form ectopic accumulations of E-Cad on the basal surface, suggesting that they are unable to form proper adherens junctions (Fig. 6G).

Taken together these data suggest that contact between stellate cells and the basal surface of tubule epithelial cells coincides with the onset of polarity in the ingressing cells and that this is a prerequisite for them to integrate properly.

We suggest that contact with the basal membrane of principal cells acts as a cue to promote the expression and localisation of basal proteins in stellate cells, allowing them to push between principal cells as epithelial contacts loosen during convergent-extension cell rearrangements. The remodelling of adherens junctions between principal cells that accompany these cell rearrangements (Bertet et al., 2004; Zallen and Wieschaus, 2004) would permit full intercalation of stellate cells, with their position in the epithelium being stabilised by the formation of stellate/principal cell zonula adherens junctions. This in turn would allow the development and stabilisation of full stellate cell polarity. Thus, the processes of integration and MET are closely interlinked.

Epithelia, such as the *Drosophila* midgut, dorsal vessel or the somatic follicle cells of the germarium, that form by MET have been described as secondary epithelia, in contrast to those that derive from the blastodermal epithelium without ever losing their polarity (Tepass, 1997). Interestingly the embryonic midgut epithelium and dorsal vessel develop without the expression of Crumbs and do not develop zonula adherens junctions (Tepass and Hartenstein, 1994b); the cells are either linked by septate junctions (Tepass and Hartenstein, 1994b) or depend on septate junction proteins to maintain tissue integrity (Yi et al., 2008). In the case of the midgut, clusters of mesenchymal precursor cells polarise with reference to underlying visceral mesodermal cells and the basement membrane that lies between them and the newly developing midgut epithelium (Tepass and Hartenstein, 1994a). The formation of the midgut epithelium in *Drosophila* depends on the interaction of endoderm and mesoderm (Tepass and Hartenstein, 1994a). Mutants for *laminin A* and *shg* have similar phenotypes in which the midgut precursors migrate over the visceral mesoderm but fail to polarise (Tepass and Hartenstein, 1994a; Yarnitzky and Volk, 1995). Laminin is required for heart, somatic muscle and gut development in the *Drosophila* embryo (Yarnitzky and Volk, 1995), suggesting that MET in these cells requires both basal and lateral adhesive cues.

At first sight the renal tubules present an intriguing mixture between primary (principal cells) and secondary (stellate cells) epithelia. However, the cues that underlie MET in tubule cells are unlike those of the midgut. In mutants for the basement membrane component Collagen IV (*vkg*) or in conditions in which the normal deposition of tubule basement membrane fails (in *pvr* mutants, Bunt et al., submitted for publication) stellate cells integrate and polarise normally (data not shown). Our evidence indicates that it is the polarised membrane domains of the epithelial cells with which they integrate that serve as cues for the axis of polarity during the MET of stellate cells, while contact with adherens junctions dictates the degree of ingression and ensures the positional integrity of cells in the epithelium.

The intercalation of cells during vertebrate gastrulation exhibits many parallels with the process we describe in the fly renal tubules. For example during zebrafish epiboly, cells with a mesenchymal morphology move from deeper layers in the embryo to intercalate between cells in the outer epithelial layer, becoming part of the epithelium. E-Cad is cell-autonomously required for intercalating cells to develop their mature shape and has a further role in stabilising their position within the epithelium. In *half-baked* (E-Cad) mutants these cells often ‘de-intercalate’ and return to deeper layers (Kane et al., 2005). In a similar way, during epidermal development in *Xenopus*, cells from the inner ectodermal layer intercalate between outer ectodermal cells in spaced arrays, before differentiating as specialised, epithelial subtypes (Stubbs et al., 2006). There are also many instances of cell ingression and MET during cell turnover and regeneration in vertebrate tissues. For example, in the intestine the stem cell zones lie deep in the crypt of Lieberkühn and their progeny migrate up the villus as a cohort before completing differentiation into the intestinal epithelium. Crypt cells express very low levels of E-Cad, the amount of this protein rising in cells as they differentiate. Interestingly the repression of E-Cad expression is required for normal migration and for the maintenance of the stem cell population (Escaffit et al., 2005). During kidney development the majority of the tubular system forms by recruitment of nephrogenic mesenchyme, which undergoes MET, integrating with the epithelial ureteric bud branches (Vize, 2003; Saxon, 1987). Although cell intercalation in this system occurs only at the junction between ureteric and mesenchymal cells, polarity cues for MET are required and could derive from epithelial ureteric bud cells. It seems likely that our demonstration that cell-neighbour polarity cues play important roles in orienting cells during MET will be widely relevant.

## 3 Experimental procedures

### 3.1 *Drosophila* stocks and genetics

The mutant strains used were *cv-c^M62^*, and *shg^G317^*. The CtB-Gal4 line (Sudarsan et al., 2002) was used to drive expression of UAS transgenes in the renal tubules, and G447.2 (Denholm et al., 2003) to drive expression in just stellate cells. The following UAS responder lines were used: UAS-CD8-GFP, UAS-Src-GFP, UAS-crb^30.12e^ (Wodarz et al., 1995) and UAS-aPKCΔN (Betschinger et al., 2003; Drier et al., 2002). For time-lapse movies, UAS-Src-GFP was crossed to CtB-Gal4. For all experiments in which stellate cells were labelled with a membrane-bound Gal4, UAS-CD8-GFP was used. To knock-down E-Cad in tubules, Byn Gal4, which is expressed in both principal and stellate cells, was used to drive five different UAS-E-Cad RNAi constructs: lines 3722R-1 and 3722R-2 from the National Institute of Genetics, Japan; and lines 8024, 27,081 and 27,082 from the Vienna *Drosophila* RNAi Center. The crosses were performed at 29 °C and lines 3722R-1 and 3722R-2 were combined with dicer 2. However, none of these combinations resulted in a significant knock-down of E-Cad proteins, which was assayed by immunostaining.

### 3.2 Immunohistochemistry

The following primary antibodies were used: rabbit anti-aPKC (1:500; Santa Cruz Biotechnology); rabbit anti-Baz (1:1000; gift from A.Wodarz); rat anti-Crb (1:500; gift from E.Knust); mouse anti-Cut (1:200; gift from I.Rebay); rat anti-dE-Cad (1:100; DCAD2, Developmental Studies Hybridoma Bank (DSHB)); mouse anti-Dlg (1:500; DSHB); goat anti-GFP (1:500; abCAM); rabbit anti-Lam (1:1000; DSHB) rabbit anti-Tsh (1:300; gift from S. Cohen). Embryos were fixed in 4% paraformaldehyde, using standard techniques. Primary incubations were performed overnight, followed by incubation with appropriate biotinylated secondaries and amplification using the Vector Elite ABC Kit (Vector Laboratories). For fluorescent double labelling, the appropriate secondary antibodies were used conjugated with FITC or Cy3 (Jackson Immunoresearch). When required, an additional amplification step using streptavidin conjugated Cy3 or FITC (Jackson Immunoresearch) was performed.

### 3.3 Image acquisition and manipulation

For transmitted-light microscopy, embryos were mounted in 1,3-diethyl-8-phenylxanthine (DPX) and viewed using a Zeiss Axioplan compound microscope at room temperature with a 20× (Pan-NeoFluar; NA 0.50; Zeiss) objective, a JVC-KY55B digital camera and Neotech Image Grabber. For confocal microscopy, embryos were mounted in Vectashield fluorescent mounting medium (Vector Laboratories) and viewed at room temperature with a 40× oil (Leica PL APO; NA 1.25) objective on a Leica SP1 or SP5 scanning laser microscope, and LSM software. Images were processed in Adobe Photoshop 7.0.

## Appendix A Supplementary data

Supplementary Movie S1 Time-lapse movie of a renal tubule undergoing convergent-extension movements in an embryo expressing UAS-Src-GFP under the control of CtB-Gal4. Total length – 30 min, frames taken every 150 s.
