# Supplementary figures and images for: Mesenchymal-to-epithelial transition of intercalating cells in Drosophila renal tubules depends on polarity cues from epithelial neighbours
Source: Mech Dev. 2010 Jul;127(7-8):345–57. doi: 10.1016/j.mod.2010.04.002 (PMC2963794; doi:10.1016/j.mod.2010.04.002)

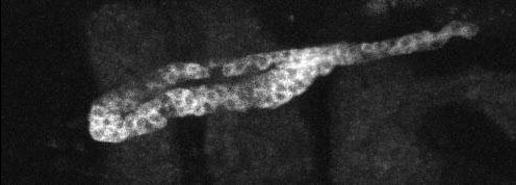

Supplement: Supplementary Movie S1 — Time-lapse movie of a renal tubule undergoing convergent-extension movements in an embryo expressing UAS-Src-GFP under the control of CtB-Gal4. Total length – 30 min, frames taken every 150 s. [file mmc1.jpg]
